# *Meloidogyne incognita* parasitism is affected by *Pseudomonas protegens* CHA0 and its effects on tomato-associated microbiota

**DOI:** 10.1186/s40793-025-00743-0

**Published:** 2025-07-01

**Authors:** Olivera Topalović, Enoch Narh Kudjordjie, Sanea Sheikh, Gnimavo Bonaventure Kenou, Frederik Bak, Flemming Ekelund, Mette Vestergård

**Affiliations:** 1 Department of Biology, Section of Terrestrial Ecology, University of Copenhagen, Copenhagen, Denmark; 2 Department of Agroecology, Section for Plant Pathology and Microbiology, Aarhus University, Slagelse, Denmark; 3 Nematology Research Unit, Department of Biology, Ghent University, Gent, Belgium; 4 Department of Plant and Environmental Sciences, University of Copenhagen, Copenhagen, Denmark; 5 Department of Environmental Science – Environmental Microbiology, Aarhus University, Roskilde, Denmark

**Keywords:** Root-feeding nematodes, *Meloidogyne*, *Pseudomonas*, 16S rRNA gene, ITS region, Plant growth, Plant-microbe interactions

## Abstract

**Background:**

The multitrophic interactions in plant rhizosphere and endosphere can be beneficial or deleterious for the plant health. The parasitism by root-feeding nematodes is on the negative end of the interaction spectrum, and may be very difficult to control. Biological agents are a promising alternative to the environmentally harmful nematicides; however, their efficiency in natural soil often seems to be low due to their limited establishment and dispersal. Thus, understanding how the introduced biological agents interact with nematodes and the surrounding microbiota is necessary to improve sustainable management of root-feeding nematodes. Here, we conducted two experiments to study the effects of *Pseudomonas protegens* strain CHA0 (CHA0) on the performance of the root-knot nematode *Meloidogyne incognita*. In the first experiment, we compared *M. incognita* performance in natural and sterilized soil in the presence and absence of CHA0. In the second experiment, we studied the composition of microbes in the rhizosphere and endosphere of tomato plants grown in native soil in response to *M. incognita* and CHA0.

**Results:**

We found that nematode performance, especially nematode reproduction, was significantly increased in native soil amended with CHA0. In addition, we found the highest relative abundance of *Pseudomonas* in tomato endosphere in response to nematode co-inoculations with CHA0, which suggests that root wounding, caused by nematodes, increased the entrance of inoculated and/or native *Pseudomonas* spp. As many *Pseudomonas* spp. are plant growth promoting, this may explain that plant growth was highest in this treatment. Furthermore, the rhizosphere of nematode-inoculated plants was enriched with *Flavobacterium*, *Hydrogenophaga* and *Variovorax*, which are genera generally associated with nematode-suppressive soils. On the other hand, other known nematode-suppressive genera such as *Bacillus*, *Lysobacter*, *Devosia* and *Rhizobium* were depleted in plants where nematodes were co-inoculated with CHA0, which may explain the higher nematode performance when plants were co-inoculated with CHA0.

**Conclusions:**

Our findings show that the effect of *P. protegens* strain CHA0 on *M. incognita* parasitism is influenced by the multitrophic interactions in the rhizosphere and endosphere of tomato plants. We must understand these interactions thoroughly to optimize sustainable means to mitigate the root-knot nematodes.

**Supplementary Information:**

The online version contains supplementary material available at 10.1186/s40793-025-00743-0.

## Background

The rhizosphere (the portion of soil influenced by the roots) is enriched with microorganisms, including bacteria and archaea, and eukaryotic fungi and protist predators, but also with microfauna such as nematodes [1]. Together these organisms in the rhizosphere make up the “rhizobiome”. The multitrophic interactions within the rhizobiome can be beneficial or detrimental for the plant. Nematodes are among the most abundant organisms in soil with estimated 4.4 ± 0.64 × 10^20^ individuals [2]. Most nematodes positively affect plant performance as through feeding on microorganisms they release the nutrients captured in microbial biomass [3] or they vector plant-beneficial microorganisms [4].

Nonetheless, the plant-pathogenic nematode counterparts include root-feeding nematodes that infect almost every crop globally [5]. Nematode feeding on the roots causes morphological deformations and disruption of the nutrient and water flow in plants. In addition, nematodes can transport secondary pathogens into the roots and form pathobiome complexes that may cause additional damage to the plants [4]. The most harmful root-feeding nematodes are root-knot nematodes (*Meloidogyne* spp.) with a damage threshold of only between one and eight eggs per cm^3^ of root material [6]. The second-stage juveniles (J2s) of the root-knot nematodes move in soil, interacting with other members of the rhizobiome. Once they enter the roots and establish feeding sites, they become sedentary. Management of root-knot nematodes is difficult due to their high population buildups and complex associations with the hosts. The restricted use of pesticides due to their negative impact on the environment has resulted in an increased research on biological agents against root-knot and other root-feeding nematodes [7, 8]. However, complex interactions with native soil microbiomes (microbiomes already present in the soil) may limit the efficiency of introduced biological agents that seem promising in simple laboratory or greenhouse experiments.

Some *Pseudomonas* species are common in the rhizobiome as biocontrol agents and plant growth promoters [9]. They produce secondary metabolites including diacetylphloroglucinol (DAPG), hydrogen cyanide, pyrrolnitrin and different modifications of pyrazine and resorcinol that act against pathogens and pests including root-knot nematodes [10, 11]. Several studies have demonstrated that *Pseudomonas protegens* (previously: *fluorescens*) strain CHA0 (CHA0) has an inhibitory effect on nematodes [12, 13]. Nonetheless, the inhibitory effects of *P. protegens* strains vary depending on the plant and nematode species, and on associations with the native soil microbiota [14, 15]. In natural soil, a DAPG-producing *P. protegens* strain Wood1R did not suppress *M. incognita* on corn, suggesting that complex interactions between this bacterial strain and the rhizobiome take place which may obstruct nematode suppression [14]. Furthermore, root-knot nematode parasitism is followed by changes in the composition of the rhizosphere and endosphere (endophytic root tissues) microbiota which may impede or facilitate nematode development [16–18]. However, the interactions between CHA0 (or other biocontrol strains) and the native microbiota in presence/absence of root-knot nematodes is poorly understood, despite the high relevance in determining the success of nematode control.

Previously, using an in vitro system, we showed that strong inter-specific interactions between CHA0 and native microbial communities in soil suspensions altered the composition of bacteria attached to the surface of the J2s of *M. incognita* [19]. Moreover, amendment of soil suspensions with CHA0 resulted in increased nematode activity [19]. Further, in a preliminary greenhouse experiment, we found that plants amended with the same CHA0 strain grew better, despite that they hosted higher numbers of J2s of *M. incognita* (data not shown). In the current study, we conducted two experiments to study the interactions between CHA0, *M. incognita* and the tomato-associated microbiota. In the first experiment, we studied if removal of the native microbial community in a soil by autoclaving would alter the effects of CHA0 on the plant-nematode association. We hypothesized that in sterilized soil, the effects of CHA0 on *M. incognita* parasitism would differ from the effects in native (unautoclaved) soil due to fewer interactions with the surrounding microbiota. In the second experiment, we used only native soil to study the effects of CHA0 on the microbial community composition in the rhizosphere and endosphere of tomato plants in the presence and absence of *M. incognita*. We hypothesized that plant growth and nematode performance correlate with the enrichment or depletion of specific taxa in these compartments.

## Materials and methods

### Experimental design and conditions

**Fig. 1:**
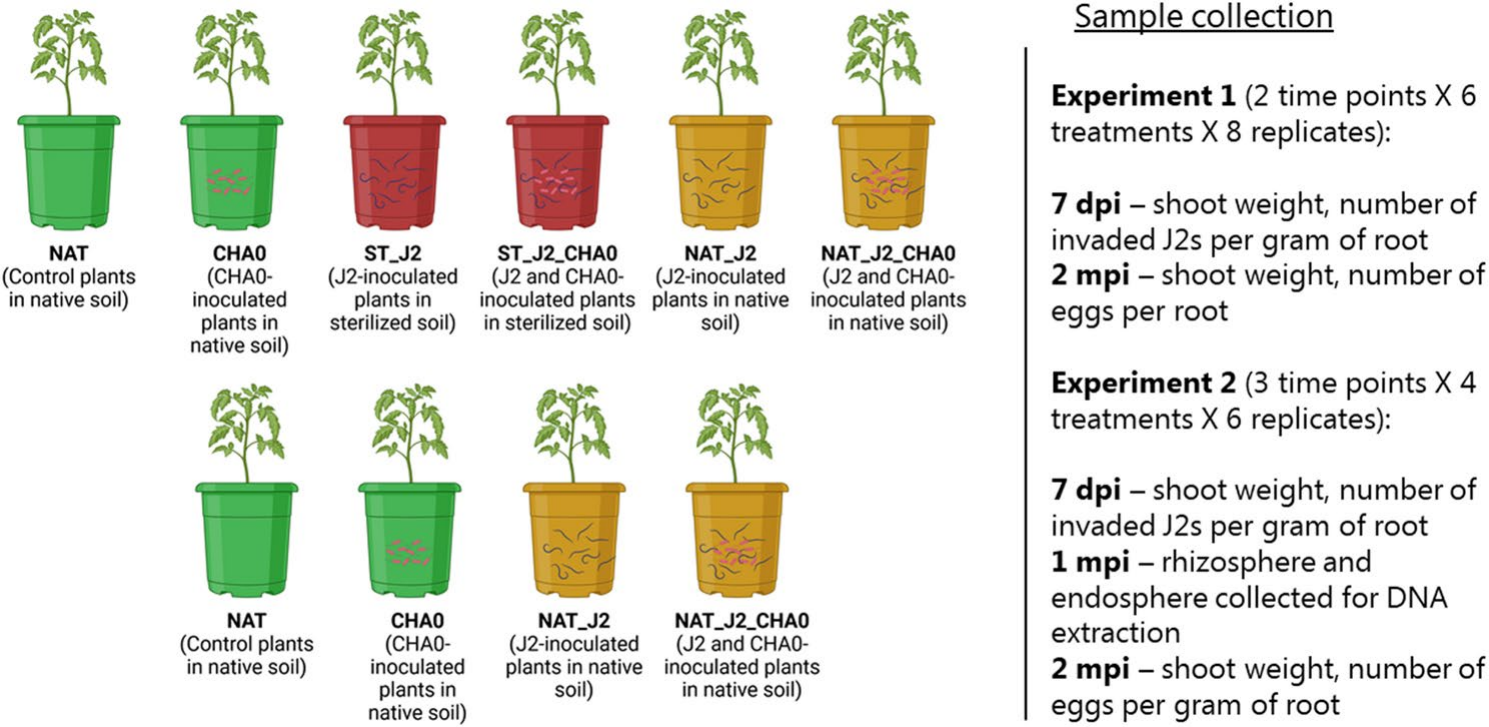
Experimental designs of the experiments in this study including the numbers of treatments, replicates, sample collection and measurements. 7 dpi stands for 7 days past J2 inoculation; 1 and 2 mpi stand for one and two months past J2 inoculation, respectively

We performed two pot experiments with tomato plants, where we studied the effects of CHA0 strain on plant growth and parasitism of the root-knot nematode *M. incognita* (Fig. 1).

In the first experiment, we compared plant and nematode performance in the presence and absence of CHA0 in sterilized (autoclaved) and native soil (see Fig. 1; Experiment 1). Our treatments were as follows: (1) Control plants in native (non-sterilized) soil (NAT); (2) Plants with CHA0 in native soil (CHA0); (3) Plants with *M. incognita* J2s in sterilized soil (ST_J2); (4) Plants with *M. incognita* J2s and CHA0 in sterilized soil (ST_J2_CHA0); (5) Plants with *M. incognita* J2s in native soil (NAT_J2); (6) Plants with *M. incognita* J2s and CHA0 in native soil (NAT_J2_CHA0). We replicated each treatment eight times.

In the second experiment, we compared plant and nematode performance in the presence and absence of CHA0 in native soil only (see Fig. 1; Experiment 2). The treatments were as follows: (1) Control plants in native soil (NAT); (2) Plants with CHA0 in native soil (CHA0); (3) Plants with *M. incognita* J2s in native soil (NAT_J2); (4) Plants with *M. incognita* J2s and CHA0 in native soil (NAT_J2_CHA0). Each treatment was replicated six times.

For both experiments, we surface-sterilized the seeds of tomato (*Lycopersicon esculentum* L. cv. Moneymaker) shaking them in 70% ethanol for 1 min and 3% NaOCl for 3 min. The surface-sterilized seeds were rinsed with sterilized water, air-dried and germinated on MS media (Murashige & Skoog, Duchefa Biochemie) for 4 days. We transplanted the four-day old tomato seedlings to pots (1 seedling per pot) containing a mixture of autoclaved sand and arable soil (1 vol:1 vol). The soil (Flakkebjerg, Denmark, 55.326°N, 11.388°E) was a sandy-clayey soil with pH of 6.8 [20]. The soil was either used as it was or autoclaved for 1 h at 121 °C before mixing with sand depending on the experiment and treatment. We used pots with 500 g of sand-soil mixture to study nematode invasion into the roots and pots with 2 kg to study nematode reproduction. We also included additional 2 kg pots in the second experiment to collect rhizosphere and endosphere samples for microbiome analyses in the different treatments one month after J2 inoculation. One week after transplanting the seedlings, we amended the plant rhizospheres with 10 ml (500 g pots) or 20 ml (2 kg pots) of a cell suspension of CHA0 (see section on strain CHA0 preparation below). After another week, we inoculated 500 *M. incognita* J2s to the 500 g pots and 2000 J2s to the 2 kg pots in 2 cm-deep holes around the stems. The pots with control plants were not inoculated with nematodes. We measured shoot weight and number of invaded J2s per gram of root seven days after J2 inoculation. Two months after nematode inoculation, we measured shoot weight and nematode reproduction (number of eggs per root (Experiment 1) and number of eggs per gram of root (Experiment 2)). The quantification of J2 invasion and reproduction as well as the preparation of J2 inocula are described in the section on nematode preparation.

### Nematode culturing, extraction from soil and root, inoculation and enumeration

To collect eggs for the preparation of the *M. incognita* J2 inoculum, we washed, excised and chopped *M. incognita* infested tomato (cv. Moneymaker) roots into smaller pieces. We then blended the chopped roots with 1% NaOCl for 15 s at low speed and 15 s at high speed in a Waring commercial blender and poured the blended suspension over a set of sieves (1 mm, 250 μm, 100 μm, and 20 μm). The eggs collected on the 20-µm sieve were washed with water extensively until the smell of bleach had disappeared. We transferred the egg suspension to a modified Baermann tray as described by Hooper et al. [21] and collected the hatched J2s on a daily basis. The first batch of hatched J2s was discarded to eliminate potential contamination with free-living nematodes originating from the roots used to obtain egg suspension. To surface sterilize nematodes, we adopted a previously published method [22]. Briefly, we incubated nematodes in a mixture of 10 mg l^− 1^ rifampicin, 25 mg l^− 1^ streptomycin-sulphate, and 10× CellCultureGuard (AppliChem, Darmstadt, Germany) for 4 h on a rotary shaker. Nematodes were washed using 3 × 25 ml of sterile tap water by centrifugation at 1000 ×g for 1 min. To study J2 invasion into the roots, 500 surface-sterilized J2s were inoculated to 500 g pots. Seven days after inoculation, we stained the nematodes in the roots with acid fuchsin [21] and counted the invaded nematodes using a dissection microscope. To study nematode reproduction, 2000 surface-sterilized J2s were inoculated to 2 kg pots. The extraction of eggs was performed as described above and the numbers were determined using a dissection microscope.

### Preparation of strain CHA0

A pre-culture of CHA0 was grown in 25 ml of 1/3 strength liquid tryptic soy broth (10 g/l, VWR135 Chemicals, Leuven, Belgium) at 28 °C overnight on a rotary shaker at 150 rpm. We used 100 µl of the pre-culture to grow the main culture in 100 ml of 1/3 strength liquid tryptic soy broth at 28 °C overnight. The bacterial cells were pelleted at 5000 ×g for 10 min and washed once using sterile tap water. We determined the bacterial optical density at 600 nm wavelength (OD_600_) using a UV-2700 spectrophotometer (Shimadzu, Kyoto, Japan). The OD_600_ was adjusted to 2 (equivalent to 10^8^ CFU ml^− 1^) to obtain the suspension used to inoculate the plant roots as described above.

### Microbiome analyses

#### Sample collection

We collected samples for microbiome analyses one month after nematode inoculation (Fig. 1). We separated the root system from the soil and removed loosely adhering soil by shaking before we transferred the roots with rhizospheres to 50 ml Falcon tubes containing 25 ml phosphate buffer saline (PBS). The content was vortexed for 30 s to disentangle rhizosphere soil from the roots. The soil suspension was centrifuged at 500×g for 5 min and the supernatant was further centrifuged at 5000×g for 10 min. The pellet was resuspended in PBS and transferred to bead-beating tubes for DNA extraction. We collected the roots from the Falcon tubes, washed and bleached them for 1 min using 1% bleach. They were washed with sterile tap water and freeze-dried for three days. The freeze-dried roots were ground using sterile steel beads in a Geno/Grinder 2010 (Ramcon, Birkerød, Denmark) to obtain the powder for DNA extraction.

#### DNA extraction, sequencing and sequence processing

DNA was extracted using DNeasy PowerLyzer PowerSoil Kit (Qiagen) according to the manufacturer’s instructions. We sent the extracted DNA, including a bacterial mock community as a positive control (ZymoBIOMICS Microbial Community DNA Standard (Zymo Research Corporation, Irvine, CA, USA)) and DNA extracted from the empty bead-beating tube as a negative control, to Novogene (UK) for amplicon sequencing of V3-V4 region of 16S rRNA gene and ITS region. The primers used for amplifying the 16S rRNA gene were 341F (CCTAYGGGRBGCASCAG) and 806R (GGACTACNNGGGTATCTAAT) [23], while the primers used for amplifying the ITS2 region were ITS3-2024F (GCATCGATGAAGAACGCAGC) and ITS4-2409R (TCCTCCGCTTATTGATATGC) [24]. The samples were sequenced on NovaSeq 6000 platform at Novogene (UK).

We determined the Amplicon Sequence Variants (ASVs) using the QIIME2 pipeline (version 2023.5.0). We removed primers from the demultiplexed paired-end reads using cutadpat plugin. We then used q2-dada2 plugin to filter and quality trim reads using default values and -p-trunc-len 0, find ASVs, remove chimeras and estimate the abundance of each ASV. For 16S rRNA gene data, taxonomic classification for the predicted ASVs was done using q2-feature-classifier plugin with pre-trained Naive Bayes classifier: “Silva 138 99% OTUs full-length sequences” downloaded from https://docs.qiime2.org/2022.2/data-resources. For the ITS region dataset, we used the 2020 release of UNITE database [25] to classify the ASVs followed by a BLAST search against nr database using default parameters for the unclassified ASVs. Bacterial ASVs with no genus level annotations were deleted in R before proceeding with the rest of the analyses.

### Statistical analysis

We analyzed nematode count data (invasion and reproduction), as well as plant shoot weight, using two-way or three-way ANOVA, where “CHA0” (presence/absence), “Nematodes” (presence/absence) and “Soil” (sterilized/native) were used as independent factors. We further used Tukey HSD posthoc test or Student’s t-test for pairwise comparisons. The homogeneity of variance was tested using Levene’s test.

Bacterial and fungal communities were analyzed and visualized in R using vegan (v2.5.7) [26], phyloseq (v1.34.0.) [27], and ggplot2 (v3.3.2) [28] packages. We removed the ASVs assigned as chloroplast or mitochondrial sequences and those unassigned at kingdom level prior to analysis. Likewise, samples with < 1,000 reads were removed from both datasets. Significant differences in the relative abundances of microbial taxa were determined using a two-group White’s nonparametric t-test in the STAMP software v2.1.3 [29, 30], with the Benjamini-Hochberg FDR to control for multiple testing. The ASV tables were transformed to relative abundances prior to beta diversity analysis by computing Bray-Curtis dissimilarity matrices and visualization, using unconstrained principal coordinates analysis (PCoA). Permutation analysis of variance (PERMANOVA) was carried out on the Bray-Curtis dissimilarity matrices to determine significant differences of experimental factors on the microbial community composition using “adonis” (with 1000 permutations) in the vegan package [26]. Additionally, we subsetted bacterial taxa that have been reported to notably correlate negatively or positively with root-feeding nematodes [19, 31], and examined their relative abundances in endosphere and rhizosphere samples and visualized with Sankey plots (https://rdrr.io/github/adrientaudiere/MiscMetabar/man/sankey_pq.html). To ensure readability of the Sankey-plots, we only plotted taxa with minimum proportion of at least 1% for the visualization.

Finally, we performed differential abundance analysis based on the negative binomial distribution to identify differentially significant microbial ASVs between experimental factors at p value < 0.01 using “DESeq2” R package [32].

## Results

**Fig. 2:**
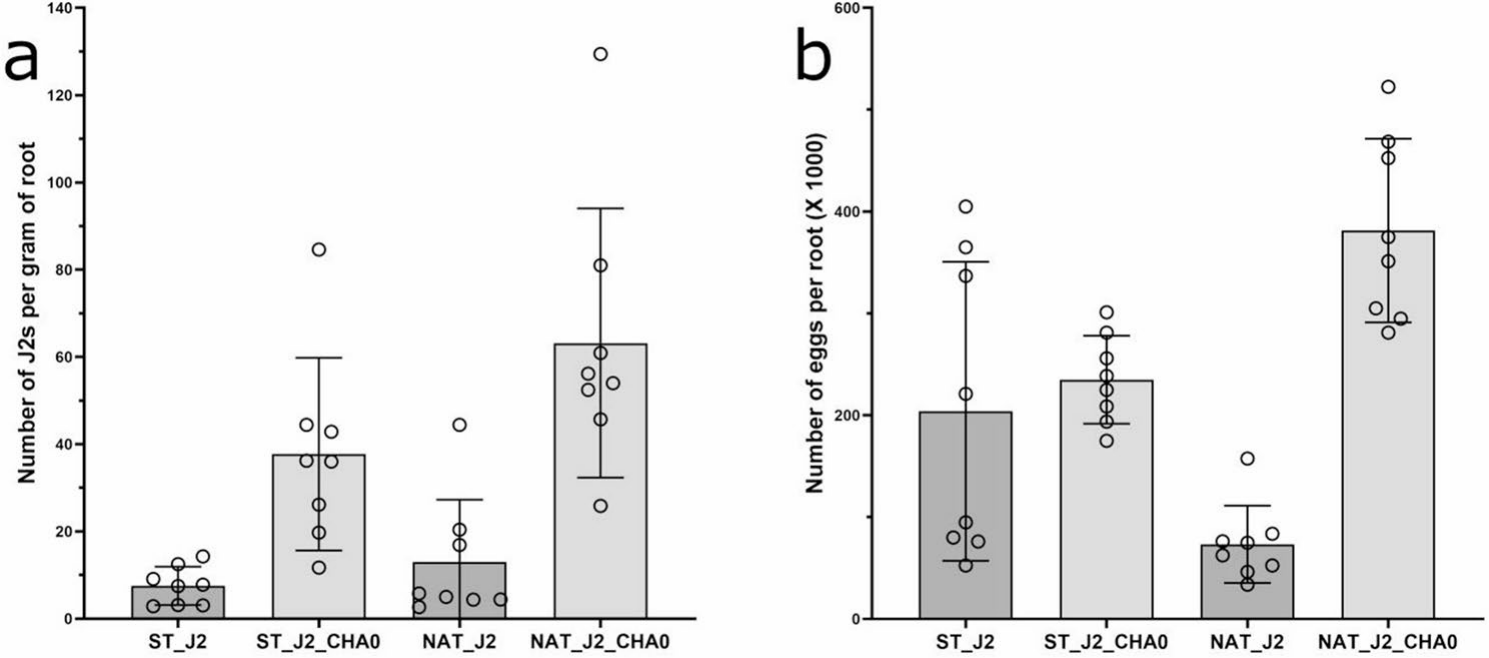
(**A**) The J2 root invasion seven days past inoculation and (**B**) nematode reproduction two months after inoculation. The treatments were as follows: ST_J2 (J2s inoculated to plants grown in sterilized soil), ST_J2_CHA0 (J2s and CHA0 inoculated to plants grown in sterilized soil), NAT_J2 (J2s inoculated to plants grown in native soil), NAT_J2_CHA0 (J2s and CHA0 inoculated to plants grown in native soil). Error bars represent standard deviation. The Two-way ANOVA and Tukey HSD postdoc tests are shown in Table S1

**Fig. 3:**
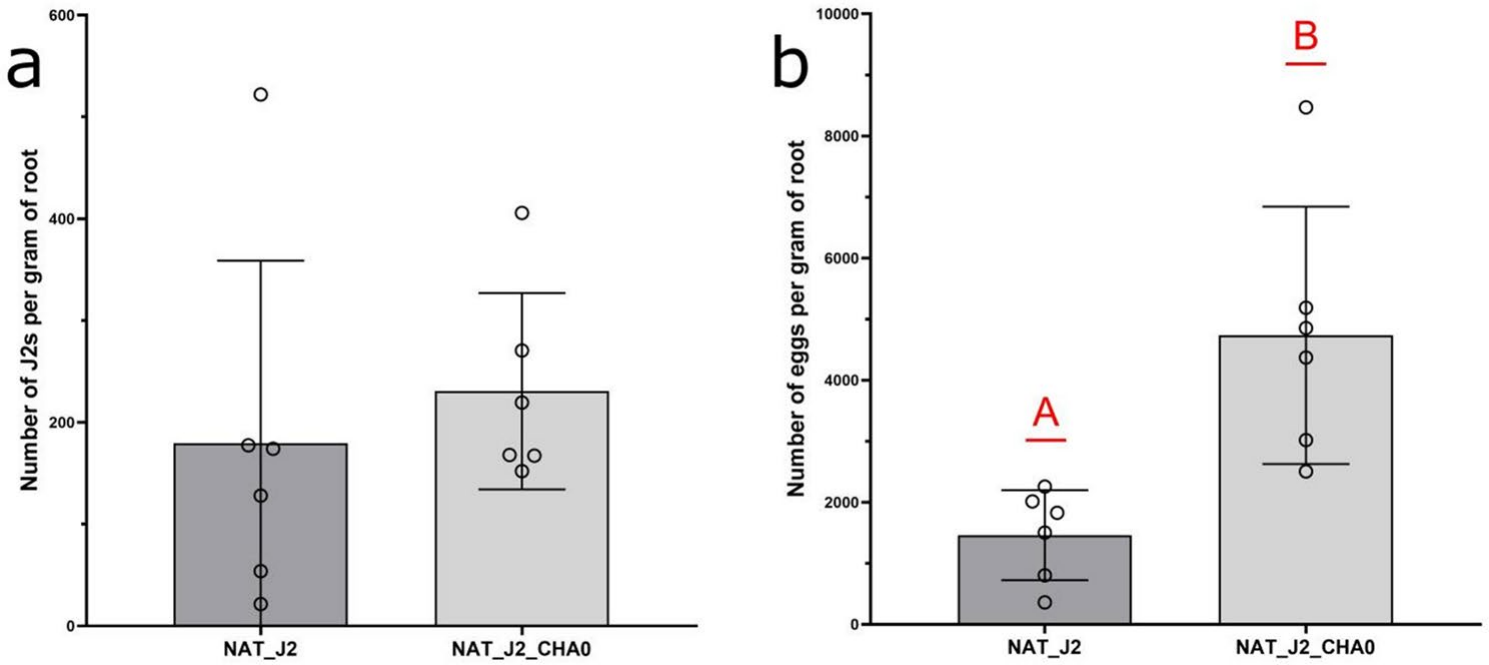
(**A**) The J2 root invasion seven days past inoculation and (**B**) nematode reproduction two months after inoculation. The treatments were as follows: NAT_J2 (J2s inoculated to the plants grown in native soil), NAT_J2_CHA0 (J2s and CHA0 inoculated to the plants grown in native soil). The letters above standard deviation bars indicate statistical difference at *p* = 0.0049 (Student’s t-test)

**Fig. 4:**
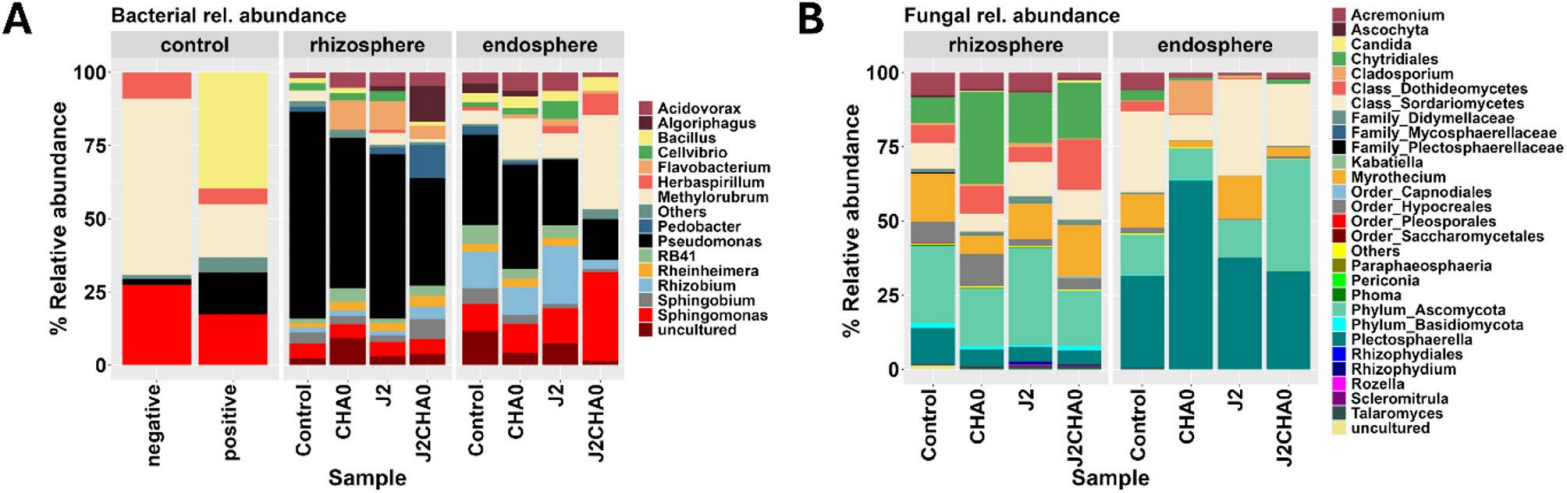
Relative abundances of bacterial (**A**) and fungal (**B**) communities in the rhizosphere and endosphere samples one month after *M. incognita* J2 inoculation (six replicates per treatment). Positive (bacterial mock community) and negative (negative control during DNA extraction) controls were included for comparison of bacterial relative abundances. “Others” comprised genera with < 1% abundance

**Fig. 5:**
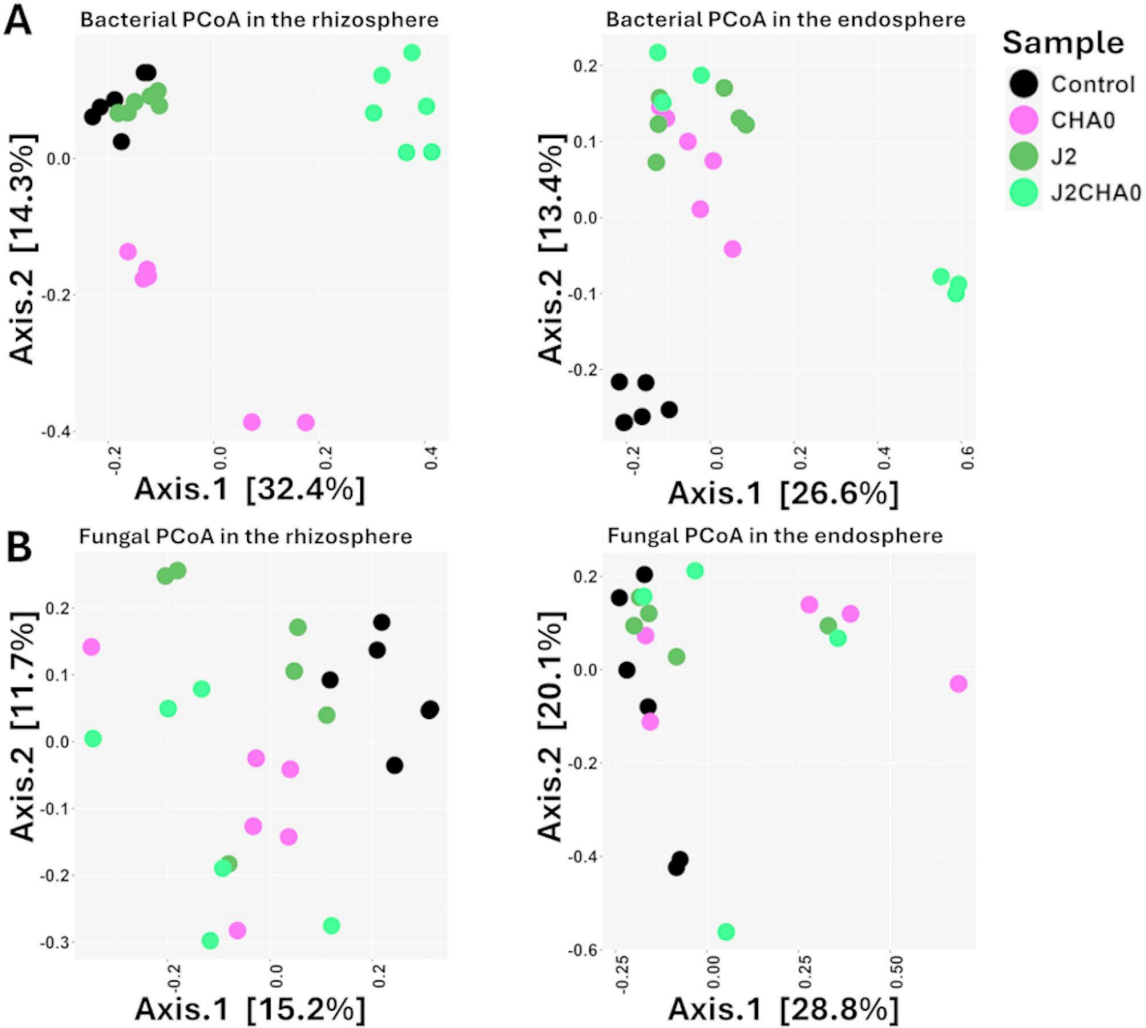
Principal coordinates analysis (PCoA) plots of bacterial (**A**) and fungal (**B**) communities in the rhizosphere and endosphere samples

**Fig. 6:**
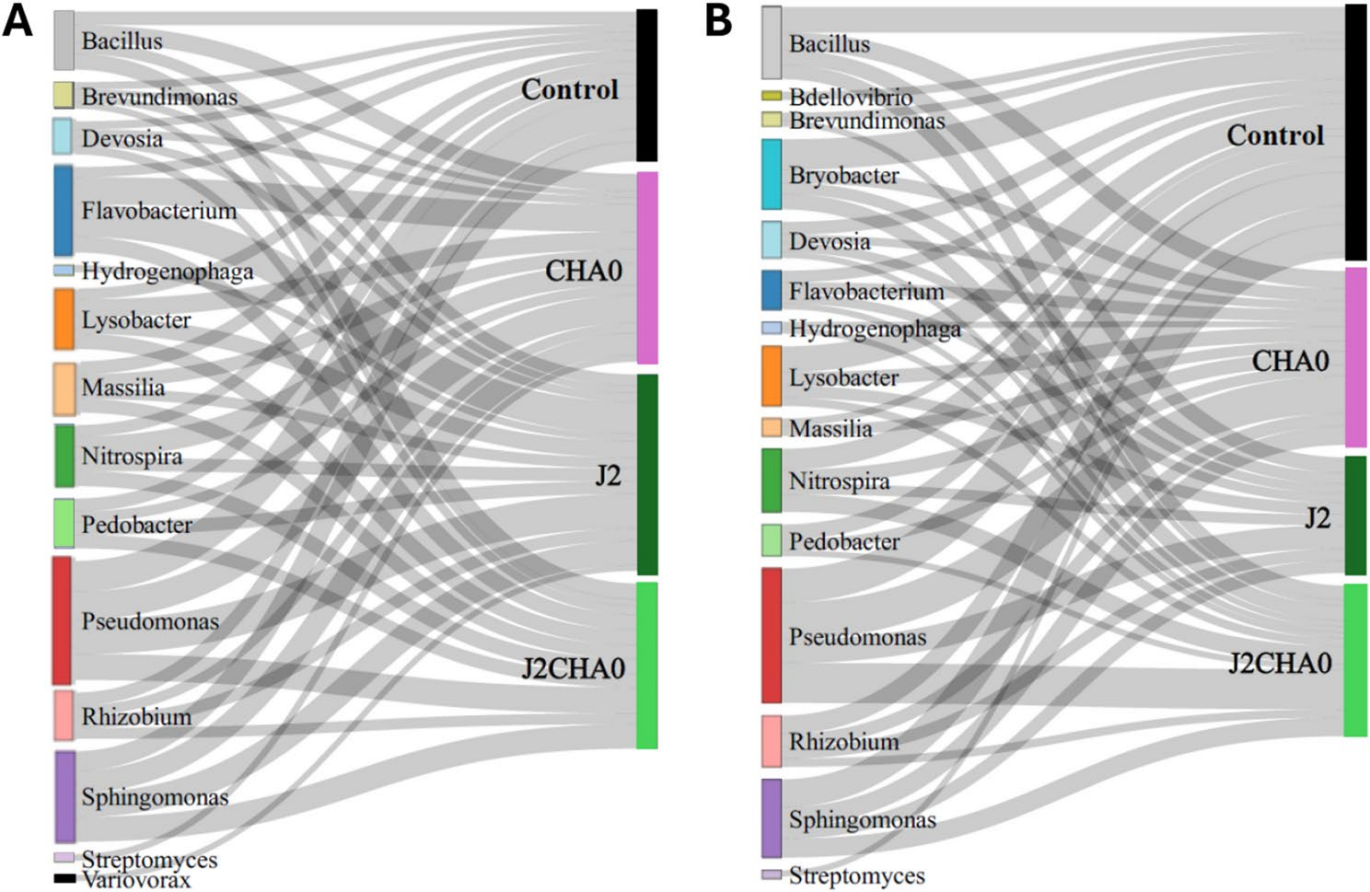
Sankey plot showing the relative abundance of bacterial taxa previously implicated in nematode suppression or facilitation in **A**) rhizosphere and **B**) endosphere samples. The colored left column represents bacterial genera, while the right column represents treatments. The width of ribbons represents the cumulative relative abundances of all ASVs within each taxonomic lineage associated with treatments

In two separate experiments, we studied the effects of CHA0 on *M. incognita* performance (invasion and reproduction) on tomato plants and tomato-associated microbiota. The results on nematode performance and microbiome analyses are provided in the main Figures (Figs. 2, 3, 4, 5 and 6), while the plant growth data and the detailed statistical analyses are provided in Supplementary Figures (Fig. S1 and S2) and Tables (Table S1 – S5). In the first experiment, we studied if presence-absence of the native soil biota would alter the effects of CHA0 on *M. incognita* parasitism and on plant growth. In the second experiment, we followed the effects of CHA0 on *M. incognita* parasitism only in native soil. Additionally, we studied how the native microbial community affected *M. incognita* parasitism in the rhizosphere and endosphere in the presence vs. absence of CHA0.

### Effects of *P. protegens* CHA0 strain on plant growth and nematode performance

#### Experiment 1

CHA0 (presence/absence) had a strong effect, while soil (sterilized/native) only had a slight effect on nematode invasion into the roots seven days past inoculation (for CHA0: *p* < 0.0001, two-way ANOVA; for Soil: *p* = 0.0407, two-way ANOVA; Table S1). CHA0 had an ameliorative effect on nematode root invasion in both native and autoclaved soil, thus the nematode-inoculated plants in the absence of CHA0 had the lowest number of invaded J2s per gram of root (treatments ST_J2 and NAT_J2, respectively; *p* < 0.0001, Tukey HSD test, Table S1) (Fig. 2a). There was no difference in J2 root invasion in treatments in native vs. autoclaved soil (for ST_J2 vs. NAT_J2, *p* = 0.9495, Tukey HSD test; for ST_J2_CHA0 vs. NAT_J2_CHA0, *p* = 0.0820, Tukey HSD test; Table S1). Two months after J2 inoculation, CHA0 had a significant effect on nematode reproduction (*p* < 0.0001, two-way ANOVA, Table S2), while soil effect was insignificant (*p* = 0.8061, two-way ANOVA, Table S2). The nematode reproduction was highest on nematode-inoculated plants in native soil amended with CHA0, where it was more than four times higher than in nematode-inoculated plants in native soil without CHA0 (*p* < 0.0001, Tukey HSD test, Table S2, Fig. 2b). Nematode reproduction was the lowest on plants inoculated with nematodes in native soil in the absence of CHA0 (Table S2, Fig. 2b). In autoclaved soil, there was no significant difference in the number of eggs between the treatments with and without CHA0 (*p* = 0.90274, Tukey HSD test, Table S2).

Seven days past J2 inoculation, soil (sterilized/native) had the strongest effect on shoot biomass (*p* < 0.0001, three-way ANOVA), which was higher in native than in autoclaved soil (Table S3, Suppl. Figure 1a). Nonetheless, CHA0 (presence/absence) had no effect (*p* = 0.3839, three-way ANOVA, Table S3), while nematodes (presence/absence) had only a slight, albeit significant, effect on shoot biomass seven days past J2 inoculation (*p* = 0.0465, three-way ANOVA, Table S3). Two months after inoculating J2s, CHA0 (presence/absence) had a strong effect on shoot biomass (*p* < 0.001, three-way ANOVA, Table S4), while soil (sterilized/native) or nematodes (presence/absence) had no effect (*p* = 0.2710, three-way ANOVA for soil; *p* = 0.8863, three-way ANOVA, for nematodes, respectively). Nematode-inoculated plants in native soil amended with CHA0 had the highest plant growth (Suppl. Figure 1b, Table S4). Shoot weight was lowest in native soil in the absence of CHA0, albeit not statistically supported.

#### Experiment 2

At seven days past J2 inoculation, nematode invasion was not different in treatments with and without CHA0 (*p* = 0.5527, t-test; Fig. 3a). Mean nematode reproduction two months after inoculation, on the other hand, was more than three times higher when the rhizosphere was amended with CHA0 (*p* = 0.0049, t-test; Fig. 3b).

In the second experiment, we did not record significant differences in the plant growth between the treatments with and without nematodes, and with and without CHA0 seven days past J2 inoculation (Suppl. Figure 2a, Table S5). Nonetheless, shoot weight in the treatment inoculated with nematodes and CHA0 two months past J2 inoculation was significantly higher than in the treatment inoculated only with nematodes (*p* < 0.0001, t-test; Suppl. Figure 2b).

### Effects of *P. protegens* CHA0 strain on the microbiomes in tomato endosphere and rhizosphere in the presence and absence of *M. incognita*

From 48 samples, we obtained 30,470 reads on average for 16S rRNA gene sequences and 19,323 reads on average for ITS sequences (Table S6). The total number of bacterial and fungal reads as well as rarefaction curves and distribution of reads between treatments and compartments (rhizosphere/endosphere) are shown in Fig. S3, S4.

#### Community composition in tomato rhizosphere and endosphere in response to *M. incognita* and *P. protegens* CHA0 strain

The bacterial relative abundances differed markedly between the rhizosphere and endosphere compartments, and across the different treatments (Fig. 4A; Fig. S5). In the rhizosphere, the relative abundance of *Pseudomonas* was significantly higher in the rhizosphere of control plants than the J2CHA0 treatment (q-value = 0.020; Fig. S5A). On the other hand, the relative abundance of *Flavobacterium* was significantly higher in the J2 or CHA0 alone treatments, while this genus was depleted in control plants (Fig. 4A; Fig. S5A). In addition, the genus *Cellvibrio* was significantly more abundant in the rhizosphere of the J2 than the J2CHA0 treatment (q-value = 0.022; Fig. S5A). We also observed the following trends, albeit statistically non-significant: the genus *Algoriphagus* was found in the rhizosphere only in the treatments with nematodes (J2 and J2CHA0), with a higher relative abundance in the treatment J2CHA0. In addition, the relative abundance of the genus *Pedobacter* appeared the highest in the rhizosphere of the treatment J2CHA0. In the endosphere, the genera *Methylorubrum* and *Sphingomonas* had the highest relative abundances in the treatment J2CHA0 compared to other treatments, albeit not statistically supported (Fig. 4A). In addition, the relative abundances of *Rhizobium* were higher in the endosphere compared to the rhizosphere compartments; they were the highest in the treatment J2 and lowest in the treatment J2CHA0. The genera *Algoriphagus*, *Pedobacter* and *Sphingobium* were significantly more abundant in the endosphere of control plants than the J2 treatment (Fig. S5B). On the other hand, the relative abundances of the genera *Acidovorax* and *Flavobacterium* were significantly higher in the endosphere of the J2 treatment than the control plants (Fig. S5B). The fungal relative abundances also differed across the treatments, most markedly in the treatment CHA0, but these differences were not statistically supported (Fig. 4B). In general, the fungal order Chytridiales and phylum Ascomycota were more abundant in the rhizosphere compartment, while the genus *Plectosphaerella* and class Sordariomycetes were more abundant in the endosphere compartment. In the rhizosphere, the relative abundance of Chytridiales was highest in the treatment CHA0. On the other hand, the CHA0 treatment had lower relative abundances of the Sordariomycetes and the genus *Myrothecium* compared to the other treatments. In the endosphere, there was a markedly higher relative abundance of *Plectosphaerella* in the CHA0 treatment, while the treatment J2CHA0 had a higher relative abundance of the phylum Ascomycota. Both treatments with CHA0 (CHA0 and J2CHA0) had reduced relative abundances of *Myrothecium* in the endosphere.

The differences in the community composition between the samples with and without nematodes and with and without CHA0 were more pronounced for bacteria than for fungi (Fig. 5). The bacterial communities associated with the J2CHA0 treatment were the most distinct in the rhizosphere compartment. In the endosphere, separation of the bacterial communities between the treatments was more distinct, although the most obvious separation was a separate clustering of the control samples. PERMANOVA analysis revealed that the sample type had a significant effect on the composition of bacterial and fungal communities (rhizosphere: bacteria, R^2^ = 0.15, *p* < 0.001; fungi, R^2^ = 0.27, *p* < 0.001; endosphere: bacteria, R^2^ = 0.35, *p* < 0.001; fungi, R^2^ = 0.23, *p* < 0.05; Table 1). Further pairwise comparisons revealed distinct significant differences of both bacterial and fungal communities in the compared treatments, except in the endosphere where fungal communities were significantly different only between CHA0 and Control (Table 1).

**Table 1:** Permutational analysis of variance (PERMANOVA) and pairwise comparisons using the “adonis” test on Bray-Curtis distance matrices for bacterial and fungal community dissimilarity assessment using 1,000 permutations

Dataset	Factors	Bacteria (16 S) *R*^2^	Fungi (ITS) *R*^2^
**Rhizosphere**			
Whole	Sample type	0.15***	0.27***
**Pairwise comparison**			
CHA0_vs_Control		0.30**	0.23**
CHA0_vs_J2		0.29**	0.18**
CHA0_vs_J2CHA0		0.42**	0.16**
Control_vs_J2		0.27**	0.22**
Control_vs_J2CHA0		0.52**	0.24**
J2_vs_J2CHA0		0.53**	0.19**
**Endosphere**			
Whole	Sample type	0.35***	0.23*
**Pairwise comparison**			
CHA0_vs_Control		0.29**	0.22**
CHA0_vs_J2		0.19**	ns
CHA0_vs_J2CHA0		0.21*	ns
Control_vs_J2		0.32**	ns
Control_vs_J2CHA0		0.33**	ns
J2_vs_J2CHA0		0.24**	ns

Significance of test indicated as ***, *p* < 0.001; **, *p* < 0.01; *, *p* < 0.05. The ns denotes not statistically significant and R^2^ is the proportion of variation explained.

#### Differential responses of bacteria to *M. incognita* and *P. protegens* CHA0 strain

Because we generally found stronger effects on the bacterial than the fungal community composition, we used DESeq analysis to determine which bacterial taxa had different abundances in the rhizosphere and endosphere compartments associated with *M. incognita* infection with and without CHA0 inoculation (Fig. S6, S7). In the rhizosphere, the genera *Asticcacaulis*, Udaeobacter and *Rubrobacter* had slightly higher abundances in the J2 treatment compared to the control plants (Fig. S6). In addition, bacterial genera *Acinetobacter*, *Helicobacter*, and *Quinella*, and families Lachnospiraceae and Muribaculaceae were more strongly associated with *M. incognita* infection alone (J2) compared to *M. incognita* co-infection with CHA0 (J2CHA0) or CHA0 infection alone (CHA0). Some genera such as *Bryobacter*, *Chitinophaga*, *Devosia*, *Dongia*, *Ferruginibacter*, *Gemmatimonas*, *Luteimonas*, *Mucilaginibacter*, *Niveispirillum*, *Peredibacter*, *Pseudoxanthomonas*, *Rhodanobacter*, and *Variovorax* were more abundant in J2CHA0 samples with a higher *M. incognita* parasitism compared to the J2 samples where the *M. incognita* parasitism was lower. In the endosphere, the J2 treatment had a higher abundance of the genus *Rheinheimera* than the control (Fig. S7). Bacterial genera *Massilia*, *Nitrospira* and *Steroidobacter* were more abundant in the J2 treatment than the J2CHA0 treatment.

### Suppressive and facilitating taxa associated with *M. incognita* parasitism in the presence and absence of *P. protegens* CHA0 strain

We further compared the relative abundances of bacterial taxa putatively implicated in the suppression or facilitation of nematode activity and/or parasitism [19, 31] across different treatments using Sankey plots (Fig. 6). In general, *Pseudomonas* had the highest relative abundance in both rhizosphere and endosphere compartments. In the rhizosphere, the highest relative abundance of *Pseudomonas* was associated with *M. incognita* infection alone (J2), followed by the control plants. Nonetheless, in the endosphere, the J2CHA0 treatment, followed by the CHA0 treatment, had the highest relative abundance of *Pseudomonas*. In addition, the rhizosphere compartment of the J2 treatment was associated with the highest relative abundance of the genus *Flavobacterium* and with the presence of the genera *Hydrogenophaga* and *Variovorax* that were absent or below detection level in the rhizosphere of other treatments. In the endosphere, *M. incognita* and/or CHA0 led to decreased relative abundances of most genera, including *Bacillus*, *Bryobacter*, *Pedobacter*, *Flavobacterium*, *Devosia*, *Rhizobium* and *Sphingomonas*. J2CHA0 had lower relative abundances of the genera *Bacillus*, *Lysobacter*, *Flavobacterium*, *Devosia* and *Rhizobium* than the other treatments, especially the control plants.

## Discussion

The success of nematode parasitism on plants is highly influenced by the multitrophic interactions between plants, nematodes and associated microbiota. The efficiency of biocontrol agents used to manage nematodes is, thus, dependent on their ability to establish in the complex soil environment and to colonize the roots [33]. *P. protegens* CHA0 strain has been reported as a nematode antagonist that synthetizes nematode-toxic compounds [12]. Nonetheless, the nematicidal activity of introduced biocontrol agents often lags behind *in situ* [14, 33]. In our previous study [19], the CHA0 strain affected the activity of *Meloidogyne* spp. J2s and the composition of microbes on the J2 surface and in the surrounding soil suspensions. In the present study, we further explored whether the parasitism of *M. incognita* on tomato plants was also influenced by this CHA0 strain and its effects on the plant microbiota.

### Effects of *P. protegens* CHA0 strain on *M. incognita* performance on plants

Comparable to our previous in vitro experiments, CHA0 facilitated nematode performance in the plant-soil system of the present study. We found that nematode invasion and reproduction were facilitated by CHA0 more pronouncedly in native than in autoclaved soil. The differences in nematode performance seemed to be positively correlated with the plant growth. CHA0 alone did not affect the plant growth, but the plants co-inoculated with *M. incognita* and CHA0 had the highest shoot biomass. Schöning and Wurst [34] showed that continuous feeding of *M. incognita* on tobacco plants increased the shoot biomass due to increased nitrogen content in shoot and root tissues. In our case, nematode infections alone decreased the shoot biomass. Moreover, CHA0 did not facilitate nematode reproduction when the native soil microbiota was eliminated by autoclaving, suggesting that CHA0 facilitated nematode reproduction indirectly via changes in the native microbiota. Thus, we further explored microbial changes associated with *M. incognita* parasitism in the presence and absence of CHA0 and the possibility for an enrichment of nematode antagonistic and plant growth promoting microbes in response to nematodes and CHA0.

### Distinct microbiomes associate with *M. incognita* parasitism in the presence and absence of *P. protegens* CHA0 strain

The rhizosphere of nematode-inoculated plants had significantly higher relative abundances of *Flavobacterium* and *Cellvibrio* compared to the control plants or plants inoculated with nematodes and CHA0. These genera may have been enriched as the antagonists or nematode facilitating bacteria as suggested previously [18, 31]. *Algoriphagus* and *Pedobacter* were enriched in the rhizosphere of nematode-inoculated plants in the presence of CHA0. We previously suggested that *Algoriphagus* can have a protective role for the nematodes as its relative abundance on active J2s was higher compared to non-motile J2s [19]. We also noticed an enrichment of *Methylorubrum* and *Sphingomonas* in the endosphere of nematode inoculated plants in the presence of CHA0. These genera were reported as antagonists of root-knot nematodes and as plant growth promoters [35, 36], and their enrichment could be linked to the increased plant growth in the nematode-inoculated plants in the presence of CHA0. *Rhizobium* was an abundant bacterial genus in the endosphere, especially in nematode-inoculated plants. Some studies point to the nematode-suppressive potential of this genus [35, 37]. On the other hand, the relative abundance of *Rhizobium* was lowest in the endosphere of nematode-inoculated plants in the presence of CHA0, which could be explained by interspecific competition between these bacteria [38]. The higher relative abundances of *Flavobacterium* and *Acidovorax* in the endosphere of nematode-inoculated plants compared to the control plants could be linked with nematode antagonistic activity of these bacteria [31]. We did not find significant differences in the fungal community composition in the rhizosphere and endosphere compartments in response to the nematode and CHA0 inoculations. However, the most distinct changes were observed in samples only inoculated with CHA0. The genus *Myrothecium*, that has a potential to antagonize nematodes [39], was depleted in the rhizosphere and endosphere of plants exposed to CHA0, but it had no apparent relation with the nematode parasitism in our study.

Differential abundance analysis revealed that the rhizosphere of nematode-inoculated plants was more enriched with the genera *Asticcacaulis*, Udaeobacter and *Rubrobacter* compared with control plants. In addition, the rhizosphere of nematode-inoculated plants had higher relative abundances of the genera *Acinetobacter*, *Helicobacter*, and *Quinella*, and families Lachnospiraceae and Muribaculaceae compared to the plants inoculated with CHA0 with or without nematodes. To our knowledge, except for *Acinetobacter* [40], none of these genera were found to be associated with nematode survival or parasitism. Some genera that were reported in association with nematode-suppressive soils, such as *Chitinophaga*, *Devosia* and *Variovorax* [31, 37, 41], were more enriched in the rhizosphere of plants co-inoculated with nematodes and CHA0 than in plants inoculated only with nematodes. It is possible that these genera had a stronger affinity to colonize the rhizosphere during the progressive nematode parasitism. On the other hand, the genus *Massilia* was more enriched in nematode-inoculated plants without CHA0. This genus has been previously detected in nematode-suppressive soils [42].

### The enrichment of nematode-suppressive bacteria is linked to the reduced *M. parasitism* in the absence of *P. protegens* CHA0 strain

We further compared the relative abundances of bacterial taxa associated with nematode suppression in different treatments. The dominant genus across all treatments was *Pseudomonas*. In the rhizosphere, the highest relative abundance of *Pseudomonas* was found for nematode-inoculated plants that had a lower nematode performance, i.e. plants inoculated with J2s only. Considering that a high abundance of *Pseudomonas* was also found for control plants without the addition of CHA0, we propose that specific nematode-suppressive strains of *Pseudomonas* already existed in the native soil. In the endosphere, the plants co-inoculated with nematodes and CHA0, followed by the plants inoculated only with CHA0, had the highest relative abundance of *Pseudomonas*. This seems consistent with the fact that nematodes create wounds while entering the roots that may have facilitated the entrance of both the inoculated and soil-native *Pseudomonas*. As many *Pseudomonas* species are plant growth promoting this may have caused the increased plant growth we observed in plants inoculated with both nematodes and CHA0. Further, our study showed that the rhizosphere of nematode-inoculated plants was enriched with nematode-suppressive genera such as *Flavobacterium*, *Hydrogenophaga* and *Variovorax* [19, 43]. On the other hand, several genera, such as *Bdellovibrio*, *Streptomyces*, *Bacillus*, *Lysobacter*, *Devosia*, and *Rhizobium* were depleted in the endosphere of plants with single or co-inoculations with nematodes and CHA0, suggesting strong nematode and CHA0 effects on the re-assembly of the endosphere microbiota in response to the external biotic stresses. Additionally, some nematode-suppressive genera such as *Bacillus*, *Lysobacter*, *Devosia* and *Rhizobium* were especially depleted in nematode-inoculated plants in the presence of CHA0, which may additionally explain a higher nematode performance in this treatment.

## Conclusions

Our study demonstrated that increased *M. incognita* parasitism on tomato plants was associated with increased plant growth in the presence of CHA0 strain. Moreover, plants co-inoculated with CHA0 and nematodes also had a higher relative abundance of *Pseudomonas* in the endosphere. The wounds created by the invading nematodes most likely enhanced the endophytic colonization of the introduced CHA0 strain and other soil-native plant-growth promoters. In addition, high *M. incognita* parasitism in the presence of CHA0 was followed by depletion of several nematode-suppressive taxa that may have diminished the resistance to *M. incognita*. Our study explores the complex interactions between introduced and native soil microbiota and how they affect nematode parasitism, which will have important implications in nematode biocontrol.

## Electronic supplementary material

Below is the link to the electronic supplementary material.

Supplementary Material 1

## Data Availability

The paired end reads generated from the 16S rRNA gene and ITS region have been deposited in the NCBI SRA repository under BioProject ID PRJNA1194017.

## Abbreviations

CHA0: *Pseudomonas protegens* strain CHA0
*M. incognita*: *Meloidogyne incognita*
J2s: Second-stage juveniles
DAPG: Diacetylphloroglucinol
dpi: Days past inoculation
mpi: Months past inoculation
16S rRNA: 16S ribosomal RNA
ITS2: Internal transcribed spacer 2
ASV: Amplicon sequence variant
ANOVA: Analysis of variance
PERMANOVA: Permutational multivariate analysis of variance
NCBI: National Center for Biotechnology Information
SRA: Sequence Read Archive
