# Supplementary material for: Meloidogyne incognita parasitism is affected by Pseudomonas protegens CHA0 and its effects on tomato-associated microbiota
Source: Environ Microbiome. 2025 Jul 1;20:79. doi: 10.1186/s40793-025-00743-0 (PMC12211340; doi:10.1186/s40793-025-00743-0)
Supplement: Supplementary file 1 — Supplementary Material 1 [file 40793_2025_743_MOESM1_ESM.docx]

**Table S1.** Two-way ANOVA analysis of the J2 root invasion 7 days past J2 inoculation with TukeyHSD posthoc test at p < 0.05.

| **J2 root invasion 7 days past J2 inoculation (Two-way ANOVA)** | | | |
| --- | --- | --- | --- |
| **Factors** | **P value** | | **F value** |
| CHA0 | 5.57e-06 ******* | | 31.099 |
| Soil | 0.0407 ***** | | 4.605 |
| CHA0*Soil | 1.926 | | 0.1761 |
| **TukeyHSD test for J2 root invasion 7 days past J2 inoculation** | | | |
| **Factors** | | **Adjusted P value** | |
| CHA0 | | 5.8e-06 ******* | |
| Soil | | 0.0407 ***** | |
| **Pairwise comparisons (CHA0*Soil)** | | | |
| No_CHA0:Native soil vs. CHA0:Native soil | | 0.00019 | |
| CHA0:Sterilized vs.CHA0:Native soil | | 0.082 | |
| No_CHA0:Sterilized soil vs. CHA0:Native soil | | 0.000045 | |
| CHA0:Sterilized soil vs. No_CHA0:Native soil | | 0.0952 | |
| No_CHA0:Sterilized soil vs. No_CHA0:Native soil | | 0.9495 | |
| No_CHA0:Sterilized soil vs. CHA0:Sterilized soil | | 0.0296 | |

Significance levels: p < 0.05 (*), p < 0.01 (**), p < 0.001 (***)

**Table S2.** Two-way ANOVA analysis of the nematode reproduction two months past J2 inoculation with TukeyHSD posthoc test at p < 0.05.

| **J2 root invasion 7 days past J2 inoculation (Two-way ANOVA)** | | | |
| --- | --- | --- | --- |
| **Factors** | **P value** | | **F value** |
| CHA0 | 0.0000129 ******* | | 27.874 |
| Soil | 0.8061 | | 0.061 |
| CHA0*Soil | 0.00018 ******* | | 18.61 |
| **TukeyHSD test for nematode reproduction 2 months past J2 inoculation** | | | |
| **Factors** | | **Adjusted P value** | |
| CHA0 | | 0.0000129 ******* | |
| Soil | | 0.8061 | |
| **Pairwise comparisons (CHA0*Soil)** | | | |
| No_CHA0:Native soil vs. CHA0:Native soil | | 0.0000013 | |
| CHA0:Sterilized vs.CHA0:Native soil | | 0.01589 | |
| No_CHA0:Sterilized soil vs. CHA0:Native soil | | 0.00285 | |
| CHA0:Sterilized soil vs. No_CHA0:Native soil | | 0.00699 | |
| No_CHA0:Sterilized soil vs. No_CHA0:Native soil | | 0.03616 | |
| No_CHA0:Sterilized soil vs. CHA0:Sterilized soil | | 0.90274 | |

Significance levels: p < 0.05 (*), p < 0.01 (**), p < 0.001 (***)

**Table S3.** Three-way ANOVA analysis of the shoot weight 7 days past J2 inoculation with TukeyHSD posthoc test at p < 0.05.

| **Shoot weight 7 days past J2 inoculation (Three-way ANOVA)** | | | |
| --- | --- | --- | --- |
| **Factors** | **P value** | | **F value** |
| CHA0 | 0.3839 | | 0.774 |
| Nematodes | 0.0465 ***** | | 4.208 |
| Soil | 0.00000408 ******* | | 28.024 |
| CHA0*Nematodes | 0.0101 ***** | | 7.264 |
| CHA0*Soil | 0.6341 | | 0.230 |
| **TukeyHSD test for shoot weight 7 days past J2 inoculation** | | | |
| **Factors** | | **Adjusted P value** | |
| No_CHA0 vs. CHA0 | | 0.3838 | |
| No_Nematodes vs. Nematodes | | 0.0465 ***** | |
| Sterilized soil vs. Native soil | | 0.0000405 ******* | |
| **Pairwise comparisons (CHA0 * Nematodes)** | | | |
| No_CHA0:Nematodes vs. CHA0:Nematodes | | 0.1204 | |
| CHA0*No:Nematodes vs. CHA0:Nematodes | | 0.9682 | |
| No_CHA0:No_Nematodes vs. CHA0:Nematodes | | 0.4471 | |
| CHA0:No_Nematodes vs. No_CHA0:Nematodes | | 0.5052 | |
| No_CHA0:No_Nematodes vs. No_CHA0:Nematodes | | 0.0088 | |
| No_CHA0:No_Nematodes vs. CHA0:No_Nematodes | | 0.3403 | |
| **Pairwise comparisons (CHA0 * Soil)** | | | |
| No_CHA0:Native soil vs. CHA0:Native soil | | 0.9633 | |
| CHA0:Sterilized soil vs. CHA0:Native soil | | 0.0257 | |
| No_CHA0:Sterilized soil vs. CHA0:Native soil | | 0.0017 | |
| CHA0:Sterilized soil vs. No_CHA0:Native soil | | 0.0655 | |
| No_CHA0:Sterilized soil vs. No_CHA0: Native soil | | 0.0053 | |
| No_CHA0:Sterilized soil vs. CHA0:Sterilized soil | | 0.8316 | |

Significance levels: p < 0.05 (*), p < 0.01 (**), p < 0.001 (***)

**Table S4.** Three-way Anova analysis of the shoot weight two months past J2 inoculation with TukeyHSD posthoc test at p < 0.05.

| **Shoot weight 2 months past J2 inoculation (Three-way ANOVA)** | | | |
| --- | --- | --- | --- |
| **Factors** | **P value** | | **F value** |
| CHA0 | 0.000782 ******* | | 13.119 |
| Nematodes | 0.8863 | | 0.021 |
| Soil | 0.271 | | 1.244 |
| CHA0*Nematodes | 0.1851 | | 1.815 |
| CHA0*Soil | 0.00266 ****** | | 10.199 |
| **TukeyHSD test for shoot weight 2 months past J2 inoculation** | | | |
| **Factors** | | **Adjusted P value** | |
| No_CHA0 vs. CHA0 | | 0.00078 ******* | |
| No_Nematodes vs. Nematodes | | 0.8864 | |
| Sterilized soil vs. Native soil | | 0.3396 | |
| **Pairwise comparisons (CHA0 * Nematodes)** | | | |
| No_CHA0:Nematodes vs. CHA0:Nematodes | | 0.003 | |
| CHA0*No:Nematodes vs. CHA0:Nematodes | | 0.8297 | |
| No_CHA0:No_Nematodes vs. CHA0:Nematodes | | 0.2059 | |
| CHA0:No_Nematodes vs. No_CHA0:Nematodes | | 0.1402 | |
| No_CHA0:No_Nematodes vs. No_CHA0:Nematodes | | 0.7187 | |
| No_CHA0:No_Nematodes vs. CHA0:No_Nematodes | | 0.7552 | |
| **Pairwise comparisons (CHA0*Soil)** | | | |
| No_CHA0:Native soil vs. CHA0:Native soil | | 0.00025 | |
| CHA0:Sterilized soil vs. CHA0:Native soil | | 0.0544 | |
| No_CHA0:Sterilized soil vs. CHA0:Native soil | | 0.0839 | |
| CHA0:Sterilized soil vs. No_CHA0:Native soil | | 0.7036 | |
| No_CHA0:Sterilized soil vs. No_CHA0: Native soil | | 0.5851 | |
| No_CHA0:Sterilized soil vs. CHA0:Sterilized soil | | 0.9983 | |

Significance levels: p < 0.05 (*), p < 0.01 (**), p < 0.001 (***)

**Table S5.** Two-way ANOVA analysis of the shoot weight 7 days past J2 inoculation with TukeyHSD posthoc test at p < 0.05.

| **Shoot weight 7 days past J2 inoculation (Two-way ANOVA)** | | |
| --- | --- | --- |
| **Factors** | **P value** | **F value** |
| CHA0 | 0.192 | 1.812 |
| Nematodes | 0.603 | 0.280 |
| CHA0*Nematodes | 0.879 | 0.130 |

**Table S6.** Mean, median and range of reads per compartment for bacterial and fungal libraries.

| whole | Mean | Median | Min | Max | Total |
| --- | --- | --- | --- | --- | --- |
| Bacteria | 30469.63 | 37973 | 2008 | 89788 | 1584421 |
| Fungi | 19323.02 | 13921.5 | 1220 | 62150 | 850213 |


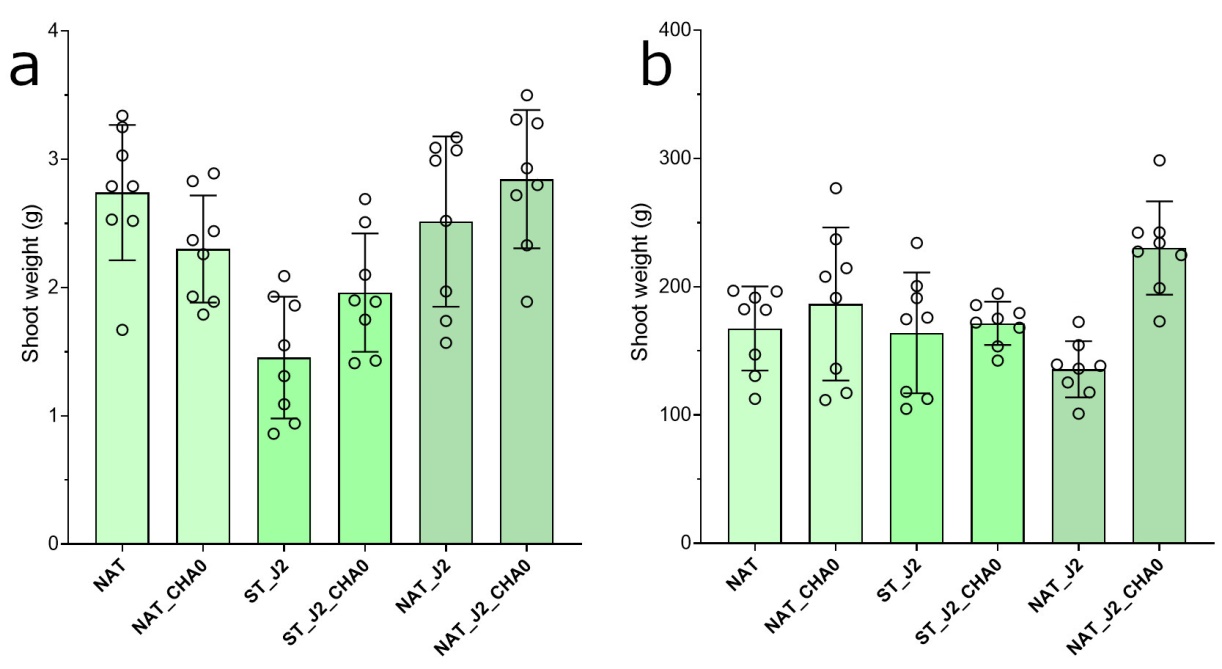


**Fig. S1. (a)** Fresh shoot weight seven days and (b) two months after J2 inoculation. The treatments were as follows: NAT (control plants grown in native soil), NAT_CHA0 (plants grown in native soil amended with CHA0), ST_J2 (J2 inoculated to the plants grown in sterile soil), ST_J2_CHA0 (J2 and CHA0 inoculated to the plants grown in sterile soil), NAT_J2 (J2 inoculated to the plants grown in native soil), NAT_J2_CHA0 (J2 and CHA0 inoculated to the plants grown in native soil). The error bars represent standard deviation. The Three-way ANOVA and Tukey HSD posthoc tests are shown in Table S3.


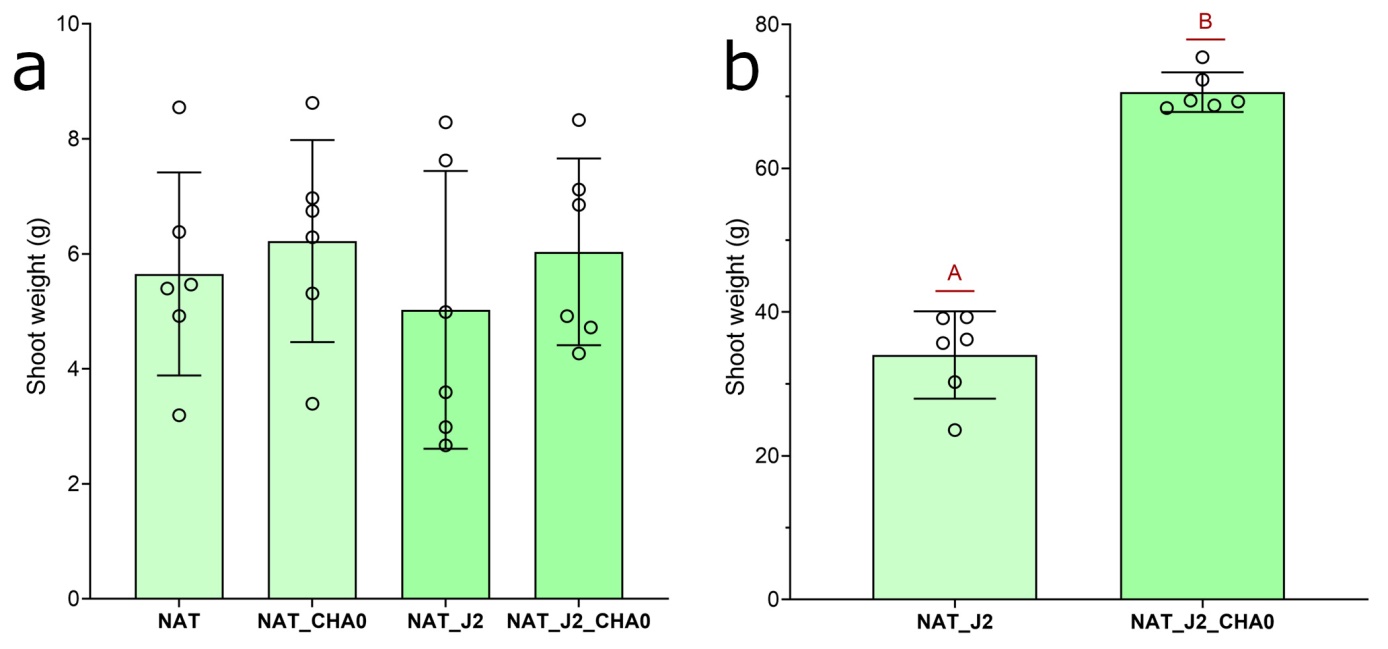


**Fig. S2.** (a) Fresh shoot weight seven days and (b) two months after J2 inoculation. The treatments were as follows: NAT (control plants grown in native soil), NAT_CHA0 (plants grown in native soil amended with CHA0), NAT_J2 (J2 inoculated to the plants grown in native soil), NAT_J2_CHA0 (J2 and CHA0 inoculated to the plants grown in native soil). The letters above standard deviation bars indicate statistical difference at p < 0.0001 (Student’s t-test). The Three-way ANOVA and Tukey HSD tests for Fig. S2.a are shown in Table S5.


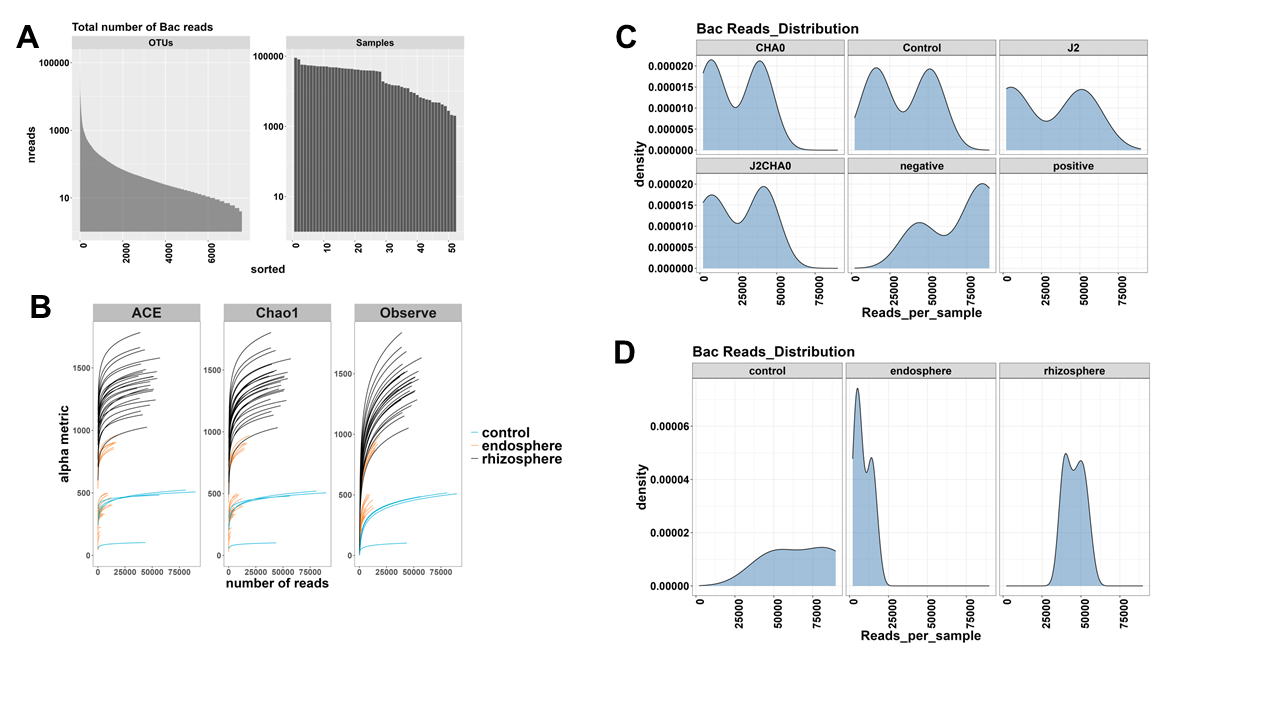


**Fig. S3.** A) Bacterial sequence reads and OTUs in samples used in this study. B) Rarefaction curves showing the sampling coverage in control, endosphere and rhizosphere. C) Bacterial read distribution in treatment samples and (D) in compartments and control.


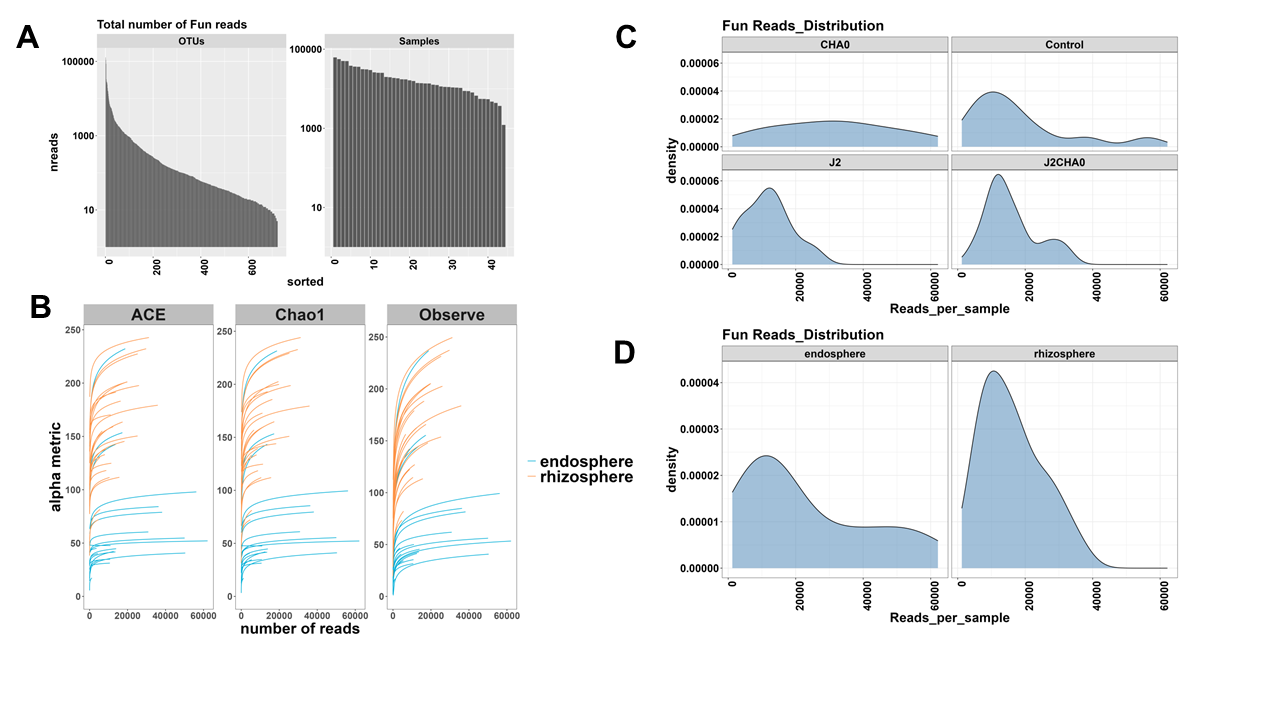


**Fig. S4.** A) Fungal sequence reads and OTUs in samples used in this study. B) Rarefaction curves showing the sampling coverage in control, endosphere and rhizosphere. C) Bacterial read distribution in treatment samples and (D) in compartments and control.

**
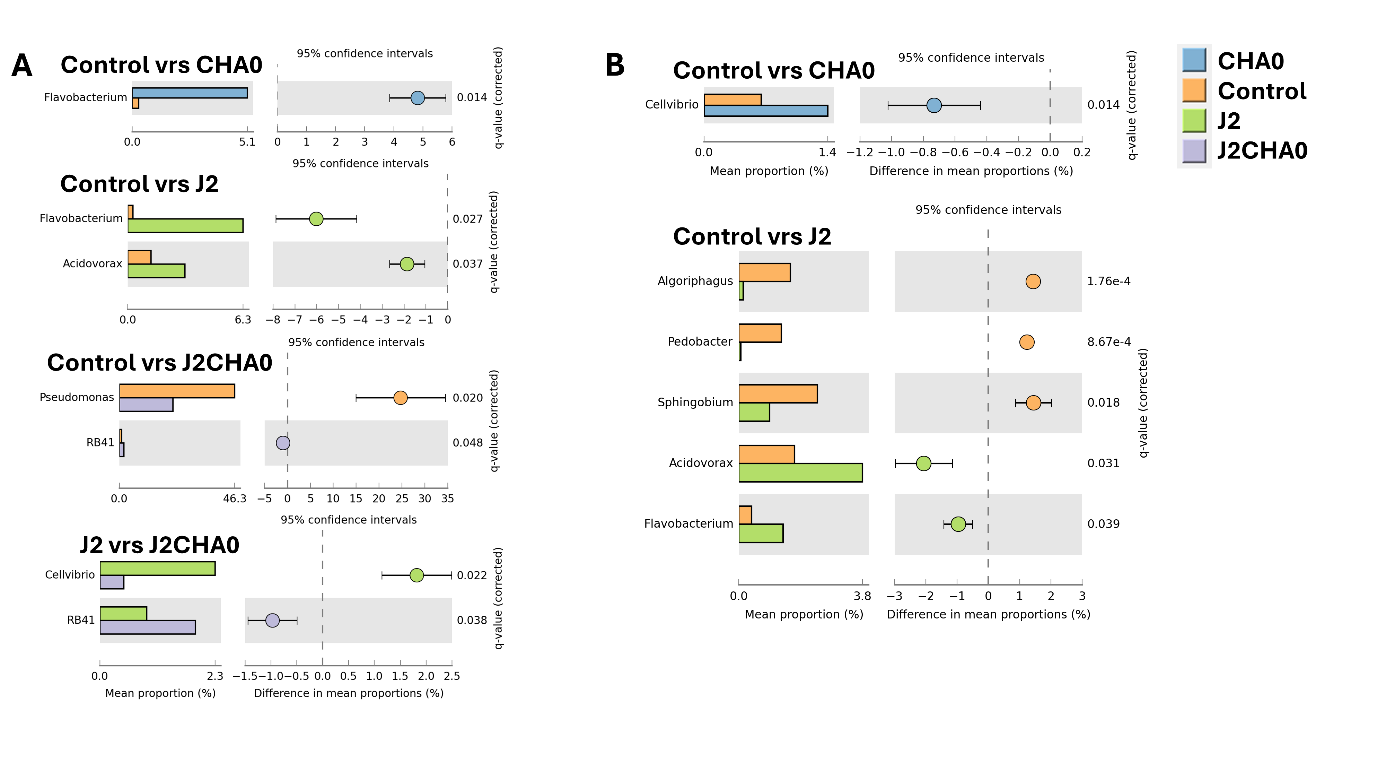
**

**Fig. S5.** Bacterial taxa in A) rhizosphere and B) endosphere that are significantly different in abundance between the sample groups.


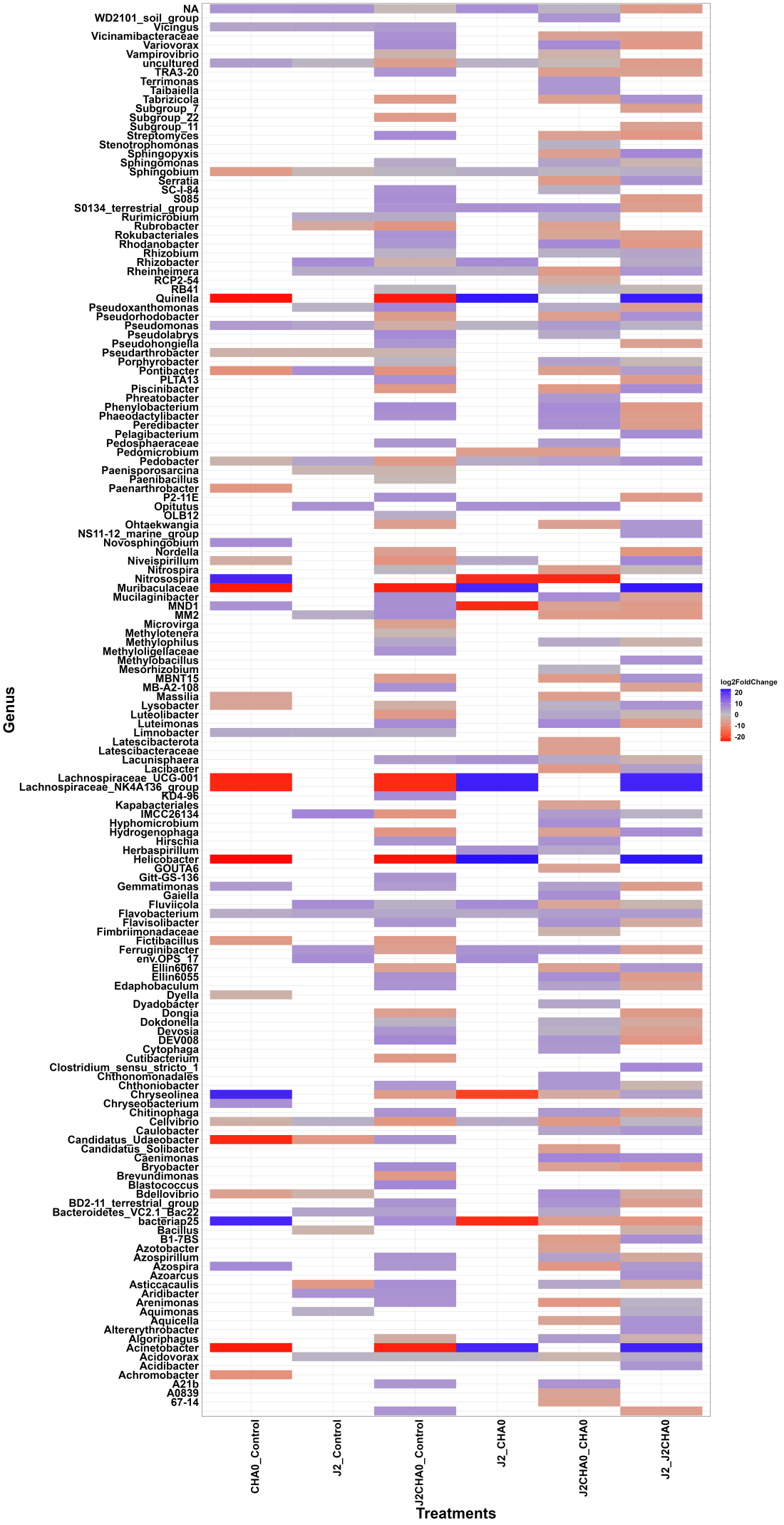


**Fig. S6.** DESeq analysis of the bacterial taxa in the rhizosphere compartment associated with different treatments. Enrichment in the compared treatment (first treatment in the label on the x-axis) are shown in red and enrichment in the reference treatment (second treatment in the label on the x-axis) are shown in blue.


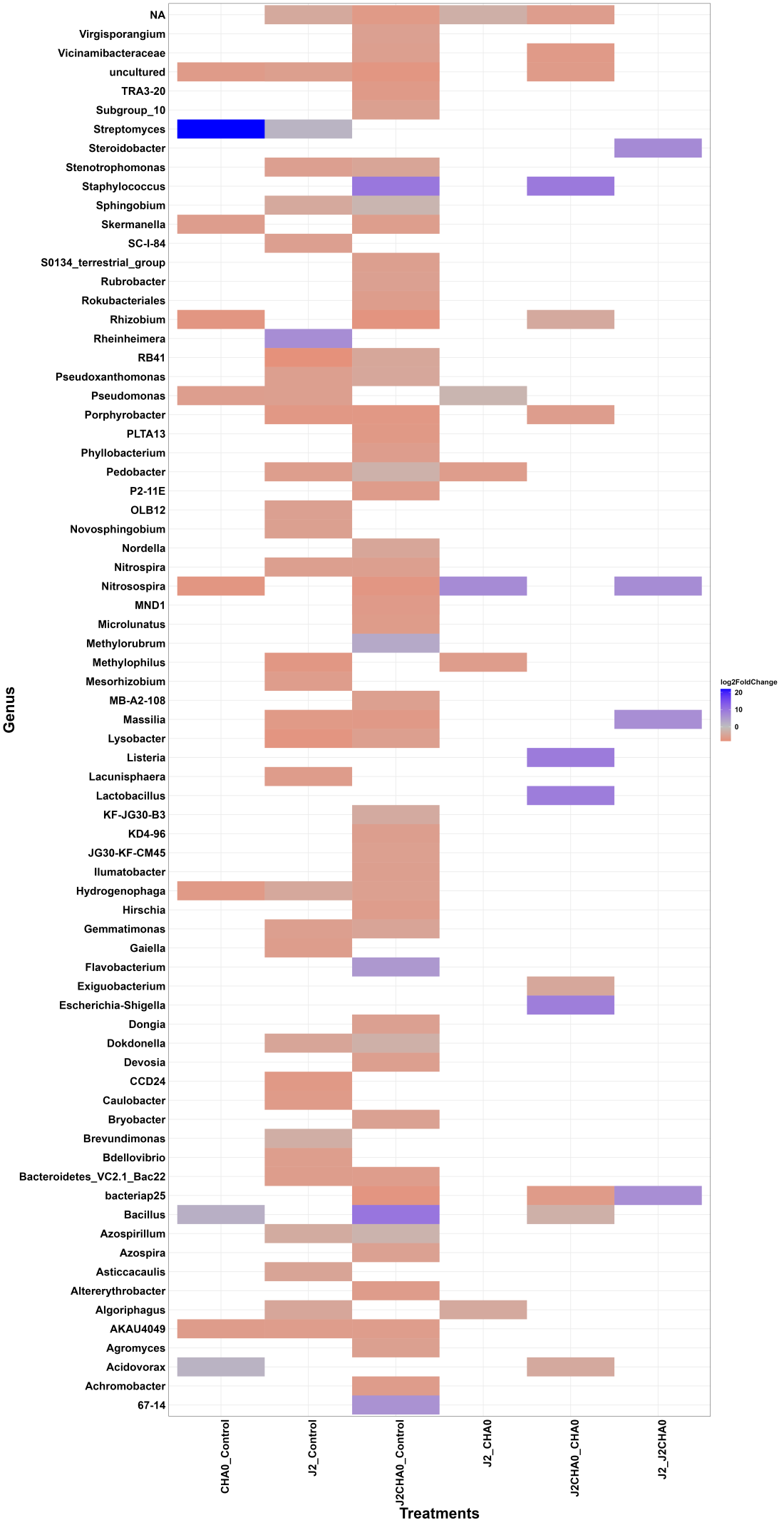


**Fig. S7.** DESeq analysis of the bacterial taxa in the endosphere compartment associated with different treatments. Enrichment in the compared treatment (first treatment in the label on the x-axis) are shown in red and enrichment in the reference treatment (second treatment in the label on the x-axis) are shown in blue.
